# Supplementary material for: An experiment on the impacts of experiential investment advice
Source: PLoS One. 2026 Feb 20;21(2):e0343126. doi: 10.1371/journal.pone.0343126 (PMC12923020; doi:10.1371/journal.pone.0343126)
Supplement: S1 File — (DOCX) [file pone.0343126.s001.docx]

**Appendix for** **An Experiment on the Intergenerational Impacts of Investment Advice**

This appendix contains additional information in support of the main paper. In Appendix I, we provide the IRB approved consent form for subjects that participated in the experiment. Appendix II shows the experiment instructions received by subjects. Appendix III provides the full regression analysis and interpretation for Experiment 1 in the main paper. Appendix IV presents the path analysis discussed in the main paper. Appendix V details the sentiment analysis on individual advice statements. Lastly, Appendix VI provides the full regression analysis and interpretation for Experiment 2 in the main paper.

**Appendix I: Consent Form**

[LARGE PUBLIC UNIVERSITY] BEHAVIORAL

INSTITUTIONAL REVIEW BOARD

CONSENT TO PARTICIPATE IN A RESEARCH STUDY

**TITLE OF STUDY:** *[Title of study removed to maintain anonymity]*

**INVESTIGATOR(S):**  *[Contact information removed to maintain anonymity]*

**PROTOCOL #: [ID removed to maintain anonymity]**

PURPOSE

You are being asked to participate in a research study. The purpose of this study is to examine how people allocate money to different investment options. We are interested in studying the factors that affect how people make investment decisions.

PARTICIPANTS

You are being asked to participate because you are an adult over 18 years of age. It is expected that there will be a total of approximately 400 participants in this study.

PROCEDURES

**Overview:**

If you choose to participate in this study, you will engage in the following task.

To begin, you will receive $5.00 as a show up fee for this experiment. This $5.00 is yours to keep and is the minimum amount you will receive.

You will then be given an additional endowment of money to begin the experiment. You will then have the opportunity to invest this money for a number of “periods”. There will be several investment opportunities available. These investments will differ in their potential return and risk.

You will choose each period how much of your money to allocate to the investments available. At the end of this experiment, you get to keep whatever money you have earned from your investing decisions. The amount you earn from your investing decisions is in addition to your $5.00 show-up fee.

**The Procedure:**

You will decide the percentage of your portfolio you wish to allocate to each investment. You will implement your allocation decisions by entering your allocation decisions into the computer program in front of you. Periodically you will be asked some questions about your beliefs. In a moment you will be given more specific information regarding the investment options and their potential return.

The amount of money you make or lose for the period will then be added to your beginning balance. This process will be repeated for the number of periods in the experiment.

To gain some experience with this procedure, you will make decisions for several practice periods. You will not be paid for these practice periods. These practice periods will not count in any way toward your performance in the experiment. After the practice periods you will have an opportunity to ask any questions.

**Time Commitment**

Each subject in the experiment will proceed at his or her own pace in making the investment allocation decisions. As advertised when you signed up, we anticipate that it should not take more than about 1 hour to complete the experiment.

DISCOMFORTS, INCONVENIENCES, AND/OR RISKS

There are no known risks from participating in this experiment.

BENEFITS

There may be no direct benefits to you as a participant in this study. However, we hope to learn about some of the underlying decision-making processes that individuals use to make investment decisions. From this research we hope to offer advice to help individuals improve their investment decisions.

CONFIDENTIALITY

# Your identity will be protected to the extent allowed by law. You will not be personally identified in any reports or publications that may result from this study. The only persons who will have access to the data from this experiment will be the principal investigators and the institution’s Institutional Review Board. The data will be stored on the computers of the principal investigators and will be kept for a maximum of ten years. All the data from this experiment will be stored separately from the consent forms of the study so that confidentiality will be protected.

The Department of Health and Human Service (HHS), other federal agencies as necessary, the [Large Public University] and the Institutional Review Board and may inspect your study records.

# COSTS/COMPENSATION

Each subject will make a series of financial decisions, and the minimum compensation is $5.00. You could earn up to $50.00 depending upon your performance in the experiment. At the end of this experiment, you will receive cash compensation for your participation. A research endowment fund given to the [Large Public University] has provided funds for this study. The names of those who receive compensation will be recorded.

# RIGHT TO REFUSE OR WITHDRAW

You may refuse to participate or withdraw from the study at any time without penalty. *If at any time you decide to withdraw, you will receive compensation of $5.00.*  If the study design or use of the data is to be changed, you will be so informed and your consent re-obtained. You will be told of any significant new findings developed during the course of this study, which may relate to your willingness to continue participation.

# QUESTIONS

If you have any questions, please ask us. If you have additional questions later, please contact:

*[Contact information removed to maintain anonymity]*

You may ask about your rights as a research subject or you may report (anonymously if you so choose) any comments, concern, or complaints to the [Large Public University] and its Institutional Review Board, telephone number [removed for anonymity], or by addressing a letter to [contact information removed for anonymity].

# CLOSING STATEMENT

I have read ( ) this consent form or have had it read to me ( ). [Check one.]

___________________ has explained the study to me and all of my questions have been answered. I have been told of the risks or discomforts and possible benefits of the study.

If I do not take part in this study, my refusal to participate will involve no penalty or loss of rights to which I am entitled. I may withdraw from this study at any time without penalty [or loss of other benefits to which I am entitled].

I have been told my rights as a research subject, and I voluntarily consent to participate in this study. I have been told what the study is about and how and why it is being done. All my questions have been answered.

I will receive a signed and dated copy of this consent form.

Signature of Participant Date

Signature of Person Obtaining Consent Date

Signature of Investigator Date

**Appendix II: Representative Condition Instructions (*Generation 2 – Negative Condition*)**

**Show-Up Fee**

You will now be given $5.00 as a show-up fee for your participation in this experiment. This show-up fee is separate compensation from your earnings in the asset allocation task. You will get to keep this show-up fee plus any earnings from the asset allocation task.

**Experimental Points**

The decisions you make in this experiment will earn you experimental points shown in €. At the end of the experiment these points will be converted into dollars at the conversion rate of €Points/100,000. For example, €100,000 points equals $1.00.

**Estimating Asset B Returns**

Please take a look at the screen in front of you. Your first task in this experiment is to estimate what you think the percentage return on Asset B will be in the next period. Each year that your estimate is within plus or minus 10% from the actual return, you will earn €25,000 points.

Each line of the spreadsheet will be explained next. The top portion of this screen reviews your estimate of the return on Asset B in the last period and compares it with the actual return.

- **Belief Data for Year:** This cell is the last period or year of the experiment. You will play two practice periods (P1 and P2) followed by a number of years. You will not be paid for your decisions or the results of the practice periods.
- **Last year you estimated the ACTUAL return on Asset B would be:** This shows your estimate of the return on Asset B for last year. A question mark is displayed for the first year when no past data exists.
- **The actual return on Asset B last year was:** This shows the actual return on Asset B last year.
- **For your estimate last year, you have earned points of:**  If your estimate of the return on Asset B last year and the actual return on Asset B last year are within 10% of each other, you will receive €25. Otherwise, you will receive nothing for your estimate last period.
- **The total points earned so far:** This is the total number of points so far for your estimates of the return on Asset B.
- **Enter Number Here (Yellow Box)** – This is where you enter your estimate for the return on Asset B next year. Please enter a number between 100 and -100. For example, if your estimate of the return on Asset B next year is 10%, please enter 10.

After you have entered your estimate for return on Asset B next year, please click the “Click on This Button” button on the spreadsheet. Another worksheet will then appear which is explained below.

**Asset Allocation Decisions**

In this experiment you will decide how to allocate points to each of the two investments each year. The spreadsheet on the computer screen in front of you is set up for you to make your allocation decisions. Please take a moment to look at this spreadsheet. Each column of the spreadsheet will be explained next.

- **Year** – This column is the current period or year of the experiment. You will play two practice periods (P1 and P2) followed by a number of years. You will not be paid for your decisions or the results of the practice periods.
- **Beginning Account Balance** – This column keeps track of the amount of points you have at the beginning of each year. To begin the experiment, you have been given an endowment of €100,000. The amount of points that you make or lose from your investment decisions each year will be added or subtracted from this balance.
- **% Asset Allocation to: Asset A/Asset B** – In these cells you will enter the percentage of your account balance (%) allocated to each investment. The spreadsheet is set up so you only have to enter your allocation for Asset B and Asset A will automatically be 100-Asset B Allocation. For example, if you wish to invest 50% of your portfolio to Asset A and 50% to Asset B, you will enter 50 in the Asset B column and the Asset A column will set to 50. Enter your allocation to Asset B as a whole number between 0-100, for example 25, 50, or 75. The cells where you should enter your decisions are highlighted in yellow. The Asset B column is the only column of the worksheet into which you will add data. *Note: To ensure that your allocations have been entered in a cell, after you typed a number in a cell be sure to hit the “Enter” key.*
- **Total Allocation (Must Sum to 100%)** – This column checks to ensure that the total amount of your allocations sums to 100%. If it does not, you will receive an error message asking you to correct your allocations.

The information on this spreadsheet will accumulate over time and will show all your past allocation decisions and the past returns on Asset A and Asset B.

**Results**

After you have made your asset allocation decision for a particular year you will then click the “Final Decision” button on the spreadsheet. Another worksheet will then appear with the results for that year. The results that you will receive include the following:

- **% Asset A/B Allocation: These** cells show your allocation to each investment.
- **Asset A/B % Return:** These cells show the actual % return on Asset A and Asset B.
- **Actual Portfolio % Return:** This cell shows that actual % return on your portfolio depending upon your allocations to Assets A and B.
- **Yearly Account** € **Return** – This is how many points you made or lost for that year depending upon your beginning account balance (BAC), your asset allocations (AA), and the actual return (AR) on each investment. The calculation in this column is: (BAC * AA Asset A * (1+AR Asset A)) + (BAC * AA Asset B * (1+AR Asset B)).
- **Ending Account Balance** € – This is the total number of €points you have at the end of the year, which is also the number of points you have to begin the next year. The calculation is: Beginning Account Balance (€) + Actual Portfolio Return (€).

The information on this spreadsheet will accumulate over time and will show all your past allocation decisions and the past returns on Asset A and Asset B.

Note the values shown on the spreadsheet will be rounded off.

**Returns on Asset A and Asset B**

The return on Asset A will be a guaranteed 3% every year.

The return on Asset B will be a random draw from the actual returns on a large well-known Stock Index in the time period from 1892-2021. The Stock Index is a portfolio of stocks made up of the largest companies in the stock market.  This is also called a Large Cap Index.  The stocks in this index include a representative sample of the leading companies in leading industries in the economy.

**How the Return on Asset B Will Be Generated.**

Each year of the experiment a random draw from a distribution of the actual returns from the Stock Index will be made to determine the annual return on Asset B. The distribution of returns and random draw will be made using the following procedure.

1. The distribution of returns on the Stock Index will be the actual real world returns on the index from a specific sequential time period. For example, you might receive the returns on the index in the time period from 1890-1925 or from 1970-2004.
2. You have been assigned a starting year sometime between 1892 and 2000. The returns on Asset B will be the returns for a consecutive time period from this starting year. For example, if your assigned starting year is 1890, you would then receive the returns from 1890-1925.
3. To understand the random draw process, imagine the following. Assume that each of the annual returns on the Stock Index from the actual time period of returns used is written on a different chip and then all of these chips are placed in a bucket. Then imagine you stuck your hand in this bucket and randomly pulled out one chip. The return written on this chip would be the return on Asset B for that year. That chip would then be set aside and another random draw from the remaining chips will take place for the next year.

To speed up this process and so that each of you can proceed at your own pace, the computer and a random number generator has been used to simulate the draw of a chip from a bucket and the order of the returns on Asset B.

To reiterate, the returns on Asset B will be actual real world returns from a well-known Stock Index from a specific sequential time period presented in a random order.

**Historical Returns on Asset B –** Below are summary statistics on the distribution of returns on the Stock Index prior to your starting year.

| Stock Index Price Returns  (not including dividends) | Statistics |
| --- | --- |
| Average | 4% |
| Standard Deviation | 19% |
| Highest Return | 46% |
| Lowest Return | -46% |

**Generational Advice**

At the end of the experiment, you will be asked to respond to the following:

- “For the next cohort that participates in this experiment, we would like to know what information you would like to pass along regarding investing in Assets A and B.

  The returns for Asset B for the next set of participants will be the Stock Index returns in the period after the period you just experienced. Imagine that you’re a parent and are passing advice to your children about investing in Asset B…”

- A sample of the information the prior cohort assessed important to pass along to you includes the following:
  - **“Investing in Asset B is dangerous as you can lose almost everything. For example, there was three-year period where if you invested €100,000 in Asset B, then you would only have €40,442 left.”**
  - **“It can be a large gamble to put a majority of your portfolio into asset B, but it sure is fun when you get a great percentage return. However, it also really hurts to lose nearly half of your portfolio in one year.”**
  - **“Asset B is extremely risky.”**

**Risk/Belief Assessment** – Periodically during the experiment you will be asked to answer some questions regarding how risky you think Asset B is your beliefs about the future returns of Asset B. These questions will include the following.

1. On a scale from 1-5 (1= Not at all risky, 5= Extremely Risk) how risky is Asset B to you?
2. Going forward, I think the AVERAGE % return on Asset B will be:
3. Going forward, I think the HIGHEST % return on Asset B will be:
4. Going forward, I think the LOWEST % return on Asset B will be:
5. Going forward, I think the percent chance that Asset B will have a return HIGHER THAN 30% is:
6. Going forward, I think the percent chance that Asset B will have a return LOWER THAN 30% is:

Please answer these questions to the best of your ability.

**Using the Spreadsheet**

The only data you need to enter on the spreadsheet are your estimates of the return on Asset B, your asset allocation decisions each year, and the risk/belief assessment questions. The spreadsheet will automatically keep track of your data and perform all calculations. Please do not attempt to use the spreadsheet for any other purposes. While the spreadsheet has been constructed to prevent any other uses, failure to adhere to this guideline may result in your dismissal from the experiment.

**Questionnaire**

After the experiment, you will be asked to fill out a questionnaire.

**Compensation**

At the conclusion of the experiment, you will be paid in cash for your participation. You will receive the amount that you have earned based upon your decisions. You will be paid individually and anonymously and no one else, other than the experimenter, will know how much you received.

**Questions**

Please ask any questions you have now.

**Appendix III: Statistical Tests Experiment 1**

*A3.1 Econometric Estimation Methods*

Different estimation methods were implemented to analyze the data. We begin by explaining the challenges with each method and techniques to overcome the limitations. The first regression specification method used is OLS, since it is useful to summarize the basic correlations present in the data. Also, OLS estimates can be used for comparison to other estimation methods that may be deemed more appropriate. OLS, however, presents a number of problems with this data including: correlated panels; a dependent variable that is bounded from above and from below (the share of stocks takes values from 0 to 100 — or from 0 to 1 in the Fractional Response Model specification); and a (potentially) truncated distribution of the share of stocks variable.

The data from the experiments exhibit positive autocorrelation from period to period. For this reason, the second estimation method used is GLS; specifically, we use Prais–Winsten regressions run under the assumption of AR(1) errors, since tests of serial correlation within panels showed the existence of AR(1)- type autocorrelation.

The third specification method used controls for the potential truncation of the dependent variables (share of stocks and beliefs) are both bounded. The share of stocks is bonded between 0 and 100. Beliefs are bounded between -100 and 100. Tobit regressions help control the potential truncation in the distribution of the stock allocation variable.

The next specification method presented is the Random Effect Model estimates, which are weighted average estimates of the purely cross-sectional estimates and the purely over-time (within) estimates. Thus, Random Effects estimates are useful as they use all the information in the data set, unlike fixed effects, for example, which use only the within variation in the data.

Next, we present estimates from the Mixed Linear Model. The MLM is an estimation method commonly used in the literature because it combines both fixed and random effects. Since our data is of an experimental nature, and hence not naturally hierarchical, it is not clear that the standard errors should be clustered at any level other than by subject. Therefore, standard errors are clustered by subject in most regression specifications. Clustering by condition is generally not appropriate for most right-hand side regressors, except in the case of the experimental treatment variables. Most right-hand side regressors lose their statistical significance if standard errors are clustered by condition. Importantly, the experimental treatment variables do not lose their statistical significance if standard errors are clustered by condition. We provide an example of that in our regression tables by clustering all standard errors by condition for all variables in the Mixed Linear Model (MLM) estimation specifications, the only specification where we cluster standard errors by condition in our regression tables. We make all regression tables in which standard errors are clustered by condition for all specifications available upon request, given space limitations.

The last specification method we present is the Fractional Response Model, given that our dependent variable ‘‘share of stocks’’ can be thought of as a fractional variable taking values between 0 and 1, we follow the recommendation of Papke and Wooldridge (1996) and present the estimates from Fractional Response Models, in line with some of the most recent literature in behavioral financial economics (Bucciol and Zarri, 2015, for instance).

To summarize our methodological discussion, there are potentially two sources of problems with the OLS estimates of the allocation to stocks including correlated panels and bounded dependent variables. The only way to know the quantitative magnitude of those potential problems is to deal with them one by one, by means of GLS and the FRM estimation methods, respectively. As the results in our regression tables below show, neither problem seems too severe, as the quantitative estimates remain quite stable across different specifications within each experimental set of data. The exception is the FRM specification which seems to suggest that the other specification methods grossly underestimate the treatment manipulation on the share of stocks --by a magnitude of about half the true effect.

We begin with the regression analysis of the participant decisions for the Positive and Negative cohorts in Generation 2. The three dependent variables in our regression analyses are: (a) the allocation to the risky Asset B in the portfolio (subjects’ stock allocations), (b) subjects self-reported, incentivized, beliefs about future stock returns; (c) Subjects’ self-reported risk attitude (risk aversion).

The explanatory variables in the econometric models include: (1) The first lag of the account balance of each subject at the beginning of each period, right before subjects report their beliefs and make their asset allocations. This variable is intended to act as a proxy for wealth effects. If left unaccounted for, these wealth effects may likely bias the other regression coefficients. In particular, the treatment effect may be biased upward if this variable is omitted; (2) age (number of years of subject), (3) sex (male=1, female=0); (4) Education: a 1-7 scale with 1 = less that high school, 7 = Doctoral degree); (5) Experience, the self-reported level of financial experience of subjects (1- 4 scale , where: 1 = No experience, 2, 4 = a lot of experience); (6) Dummy variables to identify the treatment effects for each cohort. For example, “gen2negdummy” identifies the cohort of Generation 2 receiving Negative advice from Generation 1.

Finally, we note that given endogeneity concerns (simultaneity bias issues), the ‘‘belief’’ variable regarding future risky asset returns, which subjects self-reported every period during the experimental task, is not included in the regressions in which stock allocations are the dependent variable to avoid the risk of biasing the estimates for the other explanatory variables in the regressions. If the variable “belief” is entered in stock allocation regressions, its coefficient is strongly positive and statistically significant at conventional levels in Experiment 1, which rules out the possibility that subjects were hedging their ‘bets’ (for instance, declaring optimistic beliefs and allocating most of their funds to the safe asset, for example). The inclusion of “belief” as an explanatory variable in regressions where allocations to stocks is the dependent variable does not significantly change any qualitative results; in other words, the “belief” explanatory variable does not ‘‘steal’’ explanatory power from other regressors in regressions where “share of stocks” is the dependent variable.

Under a similar rationale (i.e., simultaneity bias concerns) we also did not include the self-reported measure of risk aversion that subjects self-reported every 10 periods during the experimental task in the regressions where “stocks” is the dependent variable. When risk aversion is included as a right-hand side control variable, its coefficient is statistically significant in the range of 5%-10% (depending upon the econometric specification used) and for every point increase in risk aversion subjects reduced their stock holdings by about two percentage points.

*A3.2 Risky Asset Allocations, Regression Results Experiment 1*

We start with the regression results for the Positive and Negative cohorts in Generation 2. We first discuss the results in which the dependent variable is the allocation to the risky asset (stocks). We compare the share of stocks held by subjects who received Negative versus Positive advice from Generation. Table A3.1 summarizes the main results. The main control variables are the account balance lagged once (to control for wealth effects), age, sex, education, and investment experience. The variable Gen 2 Negative is the main regressor of interest, since it takes values equal to one if the subject received negative advice from generation 1 and zero if the subject received positive advice.

**Table A3.1 – Regression Analyses of Portfolio Allocations for Experiment 1**

Dependent Variable is Stocks

|  | (1) | (2) | (3) | (4) | (5) | (6) |
| --- | --- | --- | --- | --- | --- | --- |
| Variables | OLS | Tobit | Auto | RE | MLM | FRM |
| Gen 2 Negative | -13.73*** | -14.14** | -13.73*** | -14.57*** | -14.53*** | -0.367*** |
|  | (5.158) | (5.552) | (1.310) | (5.341) | (5.225) | (0.0346) |
|  |  |  |  |  |  |  |
| Beginning Account Balance (Lag 1) | 2.29e-05** | 2.18e-05* | 2.29e-05 | -1.53e-05** | -1.37e-05*** | 6.07e-07*** |
|  | (1.03e-05) | (1.18e-05) | (2.04e-05) | (6.98e-06) | (4.80e-06) | (1.93e-07) |
| Age | -0.315 | -0.347 | -0.315** | -0.322 | -0.329 | -0.00905 |
|  | (1.079) | (1.168) | (0.149) | (1.104) | (1.002) | (0.00664) |
| Sex | 0.100 | 0.457 | 0.100 | -0.340 | -0.346 | -0.000638 |
|  | (4.418) | (4.765) | (1.193) | (4.537) | (5.191) | (0.0351) |
| Education | 1.042 | 1.318 | 1.042*** | 1.011 | 1.019 | 0.0298* |
|  | (2.691) | (2.940) | (0.326) | (2.763) | (2.675) | (0.0176) |
| Experience | 7.161** | 7.487** | 7.161*** | 7.607** | 7.558** | 0.195*** |
|  | (3.014) | (3.273) | (0.463) | (3.093) | (3.043) | (0.0211) |
| Constant | 26.99* | 25.31 | 26.99*** | 34.73** | 34.61** | -0.609*** |
|  | (15.83) | (16.94) | (5.562) | (16.07) | (17.45) | (0.113) |
|  |  |  |  |  |  |  |
| Observations | 1,972 | 1,972 | 1,972 | 1,972 | 1,972 | 1,972 |
| R-squared | 0.119 |  | 0.119 |  |  |  |
| Number of groups |  |  |  |  | 68 |  |
| Number of subjects |  |  | 68 | 68 |  |  |

Robust standard errors in parentheses (std. errors clustered by subject except for (5), which is clustered by condition)

*** p<0.01, ** p<0.05, * p<0.

As seen in Table A3.1, the first four models show that subjects who received Negative advice held between 13 and 15 percentage points less in stocks compared to subjects who received Positive advice after controlling for wealth effects, age, sex, education and investment experience in all regression specifications. In the fractional response model (FRM), subjects receiving Negative advice reduced their share of stocks by 37 percentage points.

The robustness of regressions for stocks was tested by adding a set of additional controls to the right-hand side. In particular, the following variables were used to expand the set of controls: (i) losses (a variable measuring the monetary losses experienced by subjects each period, if any, as a proxy for loss aversion; (ii) the square of losses (to check the concavity/convexity of loss aversion and the potential for “double-down” effects in the face of large losses; (iii) gains (a variable measuring the monetary gains of subjects in each period of the investment game, if any); (iv) the square of gains (to test for the existence of diminishing returns in the domain of gains; and (v) a measure of IQ, as proxied by the sum of scores corresponding to the answers subjects provided to the three questions comprising the CRT (Cognitive Reflection Test), which they took as part of the post-experiment survey. Given space constraints we do not include the corresponding regression table but make it available upon request. Summarizing the results, in no case was the treatment effect variable (gen2negdummy) compromised in its statistical validity. Similarly, the quantitative effect remained unaffected by the introduction of the additional set of controls.

Added regressors turned out to be generally statistically significant at conventional levels (with the exception of the square of losses). The coefficients on the Prospect Theory-inspired variables (losses, square of losses, gains and square of gains) were quantitatively very small, thus not warranting inclusion in the main set of regressions. The one coefficient that was both statistically and quantitatively meaningful was the CRT measure of IQ. In this case, the coefficient was very stable quantitatively across econometric specifications, indicating that every extra point of measured IQ was related to an increase in the share of stocks of about 2.5 percentage points (with minor variation across the different econometric specifications).

*A3.3 Beliefs, Regression Results Experiment 1*

Positive and Negative advice can have a direct impact on participants stock allocations and/or can have an indirect effect through the channel of risk aversion and/or subjects’ beliefs about future stock market returns. That is, advice can directly impact stock allocations and/or can make subjects either more or less risk averse depending on the nature of the advice received, and/or more optimistic or more pessimistic, also depending on the nature of the advice received.

The literature from previous behavioral finance experiments on similar investment allocation tasks of this sort (Guerrero et al., Lejarraga et al., 2016. Safford et al., 2016, Papadovasilaki et al., 2018) have uncovered mixed results in terms of the most relevant channel of transmission from treatment manipulations to subjects’ stock allocations). Some have found beliefs to be the main driver, while others have found risk aversion to be the most relevant channel by which volatility in stock market returns are transmitted to subjects’ stock allocations. Table S1 provides a statistical summary of the relationships between stock allocations, subjects’ incentivized self-reported beliefs and subjects’ self-reported risk aversion for experiment 1 broken down by period.

As Table A3.2 makes clear (refer to test of difference in means) risk aversion turns out to be statistically insignificant at conventional levels of significance whereas beliefs turn out to be strongly statistically significant. Given the results in Table S1, we proceeded to run regressions in which belief was the dependent variable and the set of right-hand side controls used in the regressions for the share of stocks are used. Results are presented in Table A3.3.

**Table A3.2 – Descriptive Statistics Experiment 1**

|  |  | Experiment 1 | |  |  |  |
| --- | --- | --- | --- | --- | --- | --- |
| Gen 1 | AVG | AVG | AVG | Median | Median | Median |
|  | Stocks | Belief | RA | Stocks | Belief | RA |
| Yr 1 | 44.20 | 0.10 | 3.37 | 40 | 0.05 | 3 |
| Yrs 1-3 | 43.48 | 0.07 | 3.37 | 40 | 0.05 | 3 |
| YRs 4-30 | 44.40 | 0.10 | 3.49 | 40 | 0.1 | 4 |
| YRs 10-30 | 44.82 | 0.10 | 3.52 | 40 | 0.1 | 4 |
| AVG Yrs 1-30 | 44.31 | 0.10 | 3.48 | 40 | 0.1 | 3 |
| Gen2Pos | AVG | AVG | AVG | Median | Median | Median |
|  | Stocks | Belief | RA | Stocks | Belief | RA |
| Yr 1 | 54.30 | 0.14 | 3.25 | 60 | 0.065 | 3 |
| Yrs 1-3 | 60.00 | 0.15 | 3.25 | 65 | 0.08 | 3 |
| YRs 4-30 | 47.63 | 0.09 | 3.57 | 50 | 0.08 | 4 |
| YRs 10-30 | 47.52 | 0.09 | 3.66 | 50 | 0.08 | 4 |
| AVG Yrs 1-30 | 48.86 | 0.09 | 3.54 | 50 | 0.08 | 4 |
| Gen2Neg | AVG | AVG | AVG | Median | Median | Median |
|  | Stocks | Belief | RA | Stocks | Belief | RA |
| Yr 1 | 31.03 | 0.07 | 3.49 | 30 | 0.05 | 3 |
| Yrs 1-3 | 30.62 | 0.08 | 3.49 | 30 | 0.07 | 3 |
| YRs 4-30 | 30.13 | 0.06 | 3.61 | 25 | 0.06 | 4 |
| YRs 10-30 | 30.17 | 0.07 | 3.64 | 25 | 0.08 | 4 |
| AVG Yrs 1-30 | 30.18 | 0.06 | 3.59 | 25 | 0.06 | 4 |
| Differences in means | Stocks | Belief | RA |  |  |  |
| Yrs 1-30 | -18.68 | -0.03 | 0.06 |  |  |  |
| StdError | 0.84 | 0.00 | 0.03 |  |  |  |
| t-stat of diff. | 22.15*** | 6.79*** | 2.04*** |  |  |  |

**Table A3.3 – Regression Analyses of Beliefs for Experiment 1**

Dependent Variable is Belief

|  | (1) | (2) | (3) | (4) | (5) |
| --- | --- | --- | --- | --- | --- |
| VARIABLES | OLS | Tobit | Auto | RE | MLM |
| Gen 2 Negative | -0.0242** | -0.0236* | -0.0242*** | -0.0247** | -0.0247** |
|  | (0.0112) | (0.0125) | (0.00526) | (0.0114) | (0.0113) |
|  |  |  |  |  |  |
| Beginning Account Balance (Lag 1) | -9.28e-08** | -1.27e-07*** | -9.28e-08 | -1.18e-07*** | -1.18e-07*** |
|  | (3.88e-08) | (4.46e-08) | (1.16e-07) | (4.02e-08) | (3.37e-08) |
| Age | -0.00151 | -0.000849 | -0.00151 | -0.00152 | -0.00156 |
|  | (0.00188) | (0.00235) | (0.00101) | (0.00189) | (0.00216) |
| Sex | -0.0191 | -0.0279* | -0.0191** | -0.0194 | -0.0194* |
|  | (0.0131) | (0.0158) | (0.00800) | (0.0131) | (0.0112) |
| Education | 0.000559 | -0.00250 | 0.000559 | 0.000539 | 0.000621 |
|  | (0.00497) | (0.00683) | (0.00326) | (0.00499) | (0.00576) |
| Experience | -0.00242 | 0.00119 | -0.00242 | -0.00213 | -0.00192 |
|  | (0.00753) | (0.00793) | (0.00287) | (0.00756) | (0.00655) |
|  |  |  |  |  |  |
| Constant | 0.152*** | 0.155*** | 0.152*** | 0.157*** | 0.157*** |
|  | (0.0354) | (0.0388) | (0.0371) | (0.0361) | (0.0382) |
|  |  |  |  |  |  |
| Observations | 1,972 | 1,972 | 1,972 | 1,972 | 1,972 |
| R-squared | 0.017 |  | 0.017 |  |  |
| Number of groups |  |  |  |  | 68 |
| Number of subjects |  |  | 68 | 68 |  |

Robust standard errors in parentheses (std. errors clustered by subject except for (5), which is clustered by condition)

*** p<0.01, ** p<0.05, * p<0.1

As Table A3.3 shows, subjects who received negative advice from Generation 1 held beliefs that were about 2.5 percentage points lower than subjects in Generation 2 who received positive advice from Generation 1 after controlling for wealth effects, age, sex, education and investment experience in all regression specifications. Note that since the dependent variable “belief” is not bounded between 0 and 1, as was the case with the share of socks in the last column of Table A3.1, and therefore is not a fraction, we do not include the FRM estimation here.

The most optimistic subjects in Generation 2 forecasted an average return of 20% from stocks, and thus a 2-percentage point reduction equates to a 10% fall in their expectations after receiving negative advice, certainly a non-negligible effect. For subjects with expectations more in line with the historical return distribution they were provided with, the effect is, of course, larger. Note that except for wealth effects (and “sex” in two of the specifications), no other right-hand side regressor in Table A3.3 has any significant statistical effect at conventional levels. In other words, the reduction in optimism is not being modulated by education, investment experience, or age.

**Appendix IV: Does the Indirect Effect of Advice Pass Through Beliefs or Risk Aversion?**

Results from Experiments 1 and 2 show that advice from prior generations has a strong impact on the stock allocations of the current generation. In this section, we test whether the treatment effect is driven in part by changes in beliefs about stock returns or changes in risk preferences. We follow the protocol of Hayes (2013) and Hayes (2018), to test if beliefs or risk preferences mediate part of the treatment effect of negative vs positive advice on stock allocations. Specifically, we conduct bootstrap mediation with errors clustered at the subject level to control for repeated choices. Table A4.1 shows the results for Experiment 1. Columns (1)-(4) test for the role of beliefs in mediating part of the difference in stock allocations between the Negative and Positive advice conditions. Results show that Generation 2 with negative advice, held lower beliefs compared to receiving positive advice. Interestingly, there is a significant indirect effect. This suggests that part of the treatment effect is driven by changes in beliefs. These changes in beliefs are associated with lower stock allocations. In contrast, columns (5)-(8) do not find a significant indirect effect for risk aversion. That is, the effect of negative vs positive advice did not appear to significantly change risk preferences. Taken together, the analysis for Generation 2 finds some support that some of the treatment effect operated by influencing beliefs and these beliefs influenced stock choices.

Table A4.1: Mediation Analysis: Predicting Stock Allocations by Beliefs and Risk Aversion (Generation 2 Negative vs Positive)

|  | (1) | (2) | (3) | (4) | (5) | (6) | (7) | (8) |
| --- | --- | --- | --- | --- | --- | --- | --- | --- |
|  | Beliefs | Stocks | Beliefs | Stocks | Risk Aversion | Stocks | Risk  Aversion | Stocks |
| Gen 2 | -0.03*∗∗* | -16.57*∗∗∗* | -0.02*∗∗* | -12.61*∗∗∗* | 0.06 | -18.45*∗∗∗* | 0.02 | -14.39*∗∗∗* |
| Negative | (0.02) | (4.99) | (0.01) | (4.98) | (0.18) | (5.11) | (0.17) | (5.17) |
| Beliefs |  | 68.31*∗∗∗* (10.06) |  | 79.68*∗∗∗* |  |  |  |  |
| Risk |  |  |  |  |  | -4.48*∗* |  | -3.41 |
| Aversion |  |  |  |  |  | (2.35) |  | (2.53) |
| Controls | No | No | Yes | Yes | No | No | Yes | Yes |
| *N* | 2280 | 2280 | 2040 | 2040 | 2280 | 2280 | 2040 | 2040 |
| Indirect Effect |  | -2.15*∗∗* |  | -1.84*∗∗* |  | -0.27 |  | -0.05 |
|  |  | (1.06) |  | (0.86) |  | (0.87) |  | (0.64) |
| Direct Effect |  | -16.57*∗∗∗* (4.99) |  | -12.61*∗∗∗* (4.98) |  | -18.45*∗∗∗* (5.11) |  | -14.39*∗∗∗* (5.17) |
| Total Effect |  | -18.72*∗∗∗*  (5.14) |  | -14.45*∗∗∗*  (5.27) |  | -18.72*∗∗∗*  (5.19) |  | -14.45*∗∗∗*  (5.27) |

(12.40)

Bootstrapped clustered standard errors at the individual level are in parentheses and were calculated using bootstrapping with 1000 replications. Controls include Age, Sex, Education, and Experience.

*∗ p <* 0*.*10, *∗∗ p <* 0*.*05, *∗∗∗ p <* 0*.*01

Repeating the analysis for Generation 3, we restricted the data to include only subjects who received either positive advice from both prior generations or negative advice from both prior generations (Table A4.2). This allows for a cleaner comparison to the mediation analysis for Generation 2. Columns (1)-(4) in Table A4.2 show the results testing if beliefs mediated the treatment effect. The results do not find a significant relationship between positive-positive vs negative-negative advice in predicting beliefs. Additionally, there is no significant indirect effect. These results suggest that the treatment effect is not explained by the treatments changing beliefs and these beliefs leading to changes in stock allocations. In contrast, regressions (5) to (8) present some evidence that risk aversion may mediate part of the treatment effect. While specifications including no controls and controls, find a significant difference in risk aversion between positive-positive and negative-negative, the indirect effect on stock allocations is only significant when not including control variables. When controls are included the p-value for the indirect effect is no longer significant at the 10% level (p<0.106).

Table A4.2: Mediation Analysis: Predicting Stock Allocations by Beliefs and Risk Aversion (Generation 3 Negative-Negative vs Positive-Positive)

|  | (1) | (2) | (3) | (4) | (5) | (6) | (7) | (8) |
| --- | --- | --- | --- | --- | --- | --- | --- | --- |
|  | Beliefs | Stocks | Beliefs | Stocks | Risk Aversion | Stocks | Risk  Aversion | Stocks |
| Gen 3 | -0.02 | -24.14*∗∗∗* | -0.02 | -21.79*∗∗∗* | 0.50*∗∗∗* | -22.67*∗∗∗* | 0.45*∗∗* | -20.79*∗∗∗* |
| Negative-  Negative | (0.03) | (5.12) | (0.04) | (6.03) | (0.18) | (5.24) | (0.19) | (6.03) |
| Beliefs |  | 54.05*∗∗∗* (12.34) |  | 55.06*∗∗∗*  (13.10) |  |  |  |  |
| Risk Aversion |  |  |  |  |  | -4.72*∗∗* (2.26) |  | -4.83*∗∗* (2.34) |
| Controls | No | No | Yes | Yes | No | No | Yes | Yes |
| *N* | 2250 | 2250 | 2100 | 2100 | 2250 | 2250 | 2100 | 2100 |
| Indirect Effect |  | -0.92 |  | -1.15 |  | -2.37*∗* |  | -2.15 |
|  |  | (1.43) |  | (2.19) |  | (1.34) |  | (1.33) |
| Direct Effect |  | -24.14*∗∗∗* (5.21) |  | -21.79*∗∗∗* (6.03) |  | -22.67*∗∗∗* (5.24) |  | -20.79*∗∗∗* (6.02) |
| Total Effect |  | -25.04*∗∗∗*  (5.21) |  | -22.94*∗∗∗*  (6.33) |  | -25.04*∗∗∗*  (5.34) |  | -22.94*∗∗∗*  (6.20) |

Bootstrapped clustered standard errors at the individual level are in parentheses and were calculated using bootstrapping with 1000 replications. Controls include Age, Sex, Education, and Experience.

*∗ p <* 0*.*10, *∗∗ p <* 0*.*05, *∗∗∗ p <* 0*.*01

Taken together, the mediation analysis finds mixed evidence for what channels are impacting investment behavior with intergenerational advice. The results for generation 2 suggest that negative advice led to lower beliefs compared to positive advice. These belief differences may explain part of why individuals chose different stock allocations across the treatments. In contrast, when subjects received multiple generations of negative or positive advice, the mediation analysis found no support for beliefs explaining the different stock allocations across the groups. In contrast, there is some suggestive evidence that the difference in risk preferences may explain part of the treatment effect. We note that caution is warranted in interpreting tests of the risk preferences channel due to risk aversion being measured less often compared to beliefs.

**Appendix V: Content Analysis of Participant Advice**

At the completion of the investment task, participants responded to a series of survey questions. One question asked participant to provide a written response to the following: **“Please briefly explain how you formed your beliefs regarding the future returns on Asset B.”**

Our first approach to analyzing this textual data was to use the sentiment analysis tool VADER (Valence Aware Dictionary and Sentiment Reasoner). We chose VADER because it does not need any training data and can provide estimates for the limited data we have. VADER provides four sentiment scores (positive, negative, neutral, compound) on each string of belief text. The compound score is calculated by summing the positive, negative and neutral scores and then is normalized to a score between -1 and +1 (i.e., from most negative to most positive). The closer the compound score it to +1, the higher the positivity of the text.

To assess if there were any differences in scores across treatment groups, we used a one-way ANOVA and found no significant differences on the compound score across the experimental groups (*F* (6, 240) = 0.72, *p* > .05). There were also no significant differences across groups on the Negative scores (*F* (6, 240) = 0.53, *p* > .05), Neutral scores (*F* (6, 240) = 0.20, *p* > .05) or Positive scores (*F* (6, 240) = 0.39, *p* > .05).

| **Subject #** | **Condition** | **Message participant offered to pass along to next cohort.** | **Vader Score** | | | |
| --- | --- | --- | --- | --- | --- | --- |
|  |  |  | **Negative** | **Neutral** | **Positive** | **Compound** |
| 1 | Gen1 | B can either go very high or very low. Most of the time when it starts drifting up or down you know it is going to go very high or very low, once it reached a low that you think i can't get any worse than slowly start to change the portfolio allocation | 0.12 | 0.82 | 0.07 | -0.45 |
| 2 | Gen1 | I mostly "risked it for the biscuit" but I figured the more good years I had the more likely I was going to have a bad year. | 0.18 | 0.70 | 0.12 | -0.23 |
| 3 | Gen1 | Asset B seemed to have good returns for the most part until there was a few really bad crashes. This affected my beliefs about Asset B. | 0.16 | 0.61 | 0.23 | 0.36 |
| 4 | Gen1 | it was a random educate guess, looked for some sort of trend but the program is random so the trends were not very clear | 0.12 | 0.88 | 0.00 | -0.48 |
| 5 | Gen1 | My beliefs on Asset B were based off the patterns of the past. I would analyze the history of the return and made my predictions accordingly. | 0.00 | 0.91 | 0.09 | 0.36 |
| 6 | Gen1 | I never trusted B completely, so no matter what growth or decay it had showed in the previous years, I kept 35% in there to hopefully see long term growth. | 0.17 | 0.58 | 0.24 | 0.41 |
| 7 | Gen1 | I just watched the numbers and got my belief from that | 0.00 | 1.00 | 0.00 | 0.00 |
| 8 | Gen1 | i took idea from the actual asset b value | 0.00 | 0.59 | 0.41 | 0.60 |
| 9 | Gen1 | There are going to be risky periods but earning percentages are good and with a basic research on the market, you can get a nice estimate | 0.04 | 0.72 | 0.24 | 0.80 |
| 10 | Gen1 | It had drastic fells in value, and some of them were too high! It seemed it was periodically and had a tendency to go up very much and then fell drastically, so it is better to not invest much in this asset. | 0.00 | 0.82 | 0.18 | 0.83 |
| 11 | Gen1 | Asset B was very risky, and it became a complete game of luck- it was hard to estimate and allocate, especially when the range was so wide. | 0.11 | 0.72 | 0.17 | 0.46 |
| 12 | Gen1 | I tried to see if there was a pattern regarding when returns would go negative. At the beginning, it seemed like they would go negative around every four or five years. So, after 4-5 years of good returns, I would start allocating less to B in case it started going negative. | 0.18 | 0.74 | 0.09 | -0.77 |
| 13 | Gen1 | There were plenty of 5-6 year long streaks where Asset B was making a high percentage return, and I managed to capitalize on that somewhat well, but was often caught off guard when the stock would suddenly plunge. It was a high risk, high reward investment | 0.05 | 0.80 | 0.16 | 0.68 |
| 14 | Gen1 | I used my ability to look at the returns and trends of it predicting it would go up when substantially down and predicting the downfall when up a good amount. | 0.00 | 0.84 | 0.16 | 0.64 |
| 15 | Gen1 | 10% a year on investments is fine. I would rather go up steadily than fall drastically. | 0.00 | 0.89 | 0.11 | 0.20 |
| 16 | Gen1 | After the first several years, 9 times out of 10 when I put a large portion of my portfolio in Asset B, it had a negative return. So I needed to do the opposite of what I thought I should do. | 0.08 | 0.86 | 0.06 | -0.30 |
| 17 | Gen1 | I based my beliefs based on what other people have told me. I have little to no experience on investing and how to create predictions. Putting more money into something that can be risky can benefit you more because there is a higher return, compared to something with low risk and low return. Knowing that there was potential for money with asset b, I would base my decisions off of that and invest more money | 0.12 | 0.79 | 0.09 | -0.11 |
| 18 | Gen1 | To be honest, I formed my beliefs regarding the future returns on Asset B by the very recent results I've achieved. While the returns are random, in my mind, I believe that the next returns will be somewhat consistent to the previous returns I have retrieved. Ultimately, I understand what could occur, but gambled on Asset B anyways. | 0.00 | 0.89 | 0.12 | 0.73 |
| 19 | Gen1 | I formed my beliefs based on both the previous year's results as well as the fact that the returns are randomly generated from historical amounts, therefore I followed the patterns but also remained wary if it had gone for prolonged periods of high return percentages. (Much like counting cards in Blackjack) | 0.00 | 0.91 | 0.09 | 0.59 |
| 20 | Gen1 | I went off the pervious return and I rough tried to not go too far from that percentage | 0.00 | 1.00 | 0.00 | 0.00 |
| 21 | Gen1 | Previous risk and applied it towards future plays | 0.21 | 0.59 | 0.20 | -0.03 |
| 22 | Gen1 | If the percentage for asset B is in the negatives, allocate most of your money into A to save out on lost money | 0.08 | 0.71 | 0.20 | 0.53 |
| 23 | Gen1 | I didn't really know what I was supposed to be going off of so I kept guessing. | 0.00 | 1.00 | 0.00 | 0.00 |
| 24 | Gen1 | It fluctuates largely so its important to not put so much into asset B. If I felt like asset B was going to have a greater return I would only slightly increase the amount I placed into it because of the fear of losing big. | 0.10 | 0.65 | 0.25 | 0.76 |
| 25 | Gen1 | I looked at how extreme the difference in the returns I was getting in asset B and it made me realize over time it would probably even out. I was getting some great returns, then really bad ones. You can try to time it to where more money is put into asset B, but it is very hard without some luck. | 0.11 | 0.80 | 0.09 | -0.38 |
| 26 | Gen1 | I beleived that the investment would continue its trend for a few weeks or until it reached around +/- 30-40% and then flip. | 0.00 | 0.94 | 0.06 | 0.10 |
| 27 | Gen1 | I noticed the previous return. If I was off by a large margin I would be more cautious with my estimates. | 0.08 | 0.92 | 0.00 | -0.18 |
| 28 | Gen1 | I tried to keep my guesses between 10-20 since I thought it most likely to be between 0-30 and I tried to allocate more to Asset B since it at least had a chance of being more than 3%. I would rather risk more than know I was only gonna get a 3% return | 0.04 | 0.88 | 0.08 | 0.40 |
| 29 | Gen1 | I decided since the average return for asset B was only 2 percent I decided to not put more than 30% of my money into it. I then guessed the returns and how much risk I was willing to take. | 0.05 | 0.89 | 0.06 | 0.10 |
| 30 | Gen1 | I made sure to invest when it 10-30% and to invest 0% when it was negative until I was sure I was in the clear. | 0.12 | 0.66 | 0.23 | 0.36 |
| 31 | Gen1 | I made my guess based on the actual percentage from the previous round. I modified based on if the number went up or down. | 0.00 | 0.95 | 0.05 | 0.08 |
| 32 | Gen1 | Pure guessing and prediction | 0.00 | 1.00 | 0.00 | 0.00 |
| 33 | Gen1 | I tried to look at any patterns I saw and go based off that but most of the time I was playing with numbers trying to see where the sweet spot was. | 0.00 | 0.83 | 0.17 | 0.74 |
| 34 | Gen1 | I started with guessing, then chose to go up or down based on the previous answer. | 0.00 | 1.00 | 0.00 | 0.00 |
| 35 | Gen1 | purely guessed | 0.00 | 1.00 | 0.00 | 0.00 |
| 36 | Gen1 | Asset B is for risk takers and that's what it takes to earn a lot of money. Calculated risks | 0.19 | 0.71 | 0.11 | -0.18 |
| 37 | Gen1 | There is no way to form beliefs about it. Returns are completely random, and it became a guessing game of when the next big fall or rise was going to occur. | 0.07 | 0.93 | 0.00 | -0.30 |
| 38 | Gen1 | I first looked at the patterns and then estimated from there | 0.00 | 1.00 | 0.00 | 0.00 |
| 39 | Gen1 | I honestly don't know, I just predicted what will be the closest to the actual value. | 0.00 | 0.72 | 0.28 | 0.66 |
| 40 | Gen2Neg | I felt it would slowly go up then crash like a wave or your winning chance on an arcade claw machine. | 0.10 | 0.62 | 0.29 | 0.64 |
| 41 | Gen2Neg | I based my beliefs on the behavior of the stock in the past few years. Also, I considered that periods with high returns would almost surely have lower returns the next year, and the same goes for periods of losses. | 0.11 | 0.84 | 0.06 | -0.26 |
| 42 | Gen2Neg | should invest more on Asset B | 0.00 | 0.64 | 0.36 | 0.42 |
| 43 | Gen2Neg | I kind of looked at the patter that was happening and the trends. When it started to decrease it would decrease a lot but then out of nowhere it would shoot up. | 0.00 | 0.92 | 0.08 | 0.37 |
| 44 | Gen2Neg | I figured that with a certain amount of large positive returns, a decent amount of negative returns had to pop up at some point. Asset B did have a good amount of large returns, so I didn't find it too risky to put a lot of my money into that. | 0.09 | 0.73 | 0.18 | 0.68 |
| 45 | Gen2Neg | since I can keep getting money on asset A as I know, I would reduce risk and put money by the last year result. | 0.08 | 0.83 | 0.09 | 0.10 |
| 46 | Gen2Neg | Probability and business cycles. I anticipated 50% of the years having positive portfolio growth rates. | 0.00 | 0.68 | 0.32 | 0.74 |
| 47 | Gen2Neg | I would look at the percent from the pervious year and would try to guess around that number. | 0.00 | 0.93 | 0.07 | 0.08 |
| 48 | Gen2Neg | Not very consistent in the long term, very risky asset | 0.16 | 0.62 | 0.22 | 0.17 |
| 49 | Gen2Neg | I believe it was mostly luck involved, but if you received a few years straight of negative returns, you're bound to get some positive returns soon. | 0.14 | 0.61 | 0.26 | 0.49 |
| 50 | Gen2Neg | When B hits to 30. it is likely to go down next year | 0.00 | 1.00 | 0.00 | 0.00 |
| 51 | Gen2Neg | return rates have a cycle | 0.00 | 1.00 | 0.00 | 0.00 |
| 52 | Gen2Neg | I put in numbers close to the previous returns to be safe | 0.00 | 0.79 | 0.21 | 0.44 |
| 53 | Gen2Neg | I based my beliefs on the information provided about the average returns of Asset B, and knew that I had a better change on average of making higher returns than with the constant 3% of Asset A. | 0.00 | 0.81 | 0.19 | 0.78 |
| 54 | Gen2Neg | There were some really high and some really low returns. | 0.21 | 0.79 | 0.00 | -0.34 |
| 55 | Gen2Neg | Asset B had a mixed performance, but outperformed the 3% returns of asset A consistently. | 0.00 | 0.72 | 0.28 | 0.61 |
| 56 | Gen2Neg | Historical return is not a true basis for return in the future | 0.18 | 0.83 | 0.00 | -0.33 |
| 57 | Gen2Neg | pick relatively close to previous year | 0.00 | 1.00 | 0.00 | 0.00 |
| 58 | Gen2Neg | I saw a pattern of returns exploding after 3 years of negative returns generally leading to 2 - 3 years of positive returns | 0.13 | 0.74 | 0.13 | -0.03 |
| 59 | Gen2Neg | I noticed small patterns within the Asset and anticipated the growth or crash. | 0.15 | 0.56 | 0.29 | 0.34 |
| 60 | Gen2Neg | I noticed that around every 3 years it would take a dive, so about every 3 years I would invest less in Asset B; but also because I was guaranteed a 3% return on asset a I safely decided to invest more in that than asset b (I'm a graduate student I look for stability) | 0.00 | 0.80 | 0.20 | 0.91 |
| 61 | Gen2Neg | I would just guess and not be too risky all the time. I would stay within range of the last number and occasionally spike the number to be risky. Mostly though, I stayed within +/- 15 range of the last number. | 0.04 | 0.84 | 0.12 | 0.11 |
| 62 | Gen2Neg | I always based it off previous years and would keep it between 10 over or under. | 0.00 | 1.00 | 0.00 | 0.00 |
| 63 | Gen2Neg | I formed my beliefs based on probabilities. If returns were high for consecutive years, then I allocated less and less & vice versa | 0.00 | 1.00 | 0.00 | 0.00 |
| 64 | Gen2Neg | Even if it is risky you can make a lot of money | 0.14 | 0.86 | 0.00 | -0.20 |
| 65 | Gen2Neg | Watching the patterns | 0.00 | 1.00 | 0.00 | 0.00 |
| 66 | Gen2Neg | i just believed it would either constantly go down and dip up or constantly go up but dip down a bit | 0.00 | 1.00 | 0.00 | 0.00 |
| 67 | Gen2Neg | Asset B was incredibly volatile, shifting from double digit positive returns to double digit negative returns in the following year. Unless I had a long period of time to invest this money in to afford the risk, I would not subject my money to that much volatility. | 0.11 | 0.78 | 0.11 | 0.08 |
| 68 | Gen2Neg | I tried to follow previous patterns and I also tried to rely on my gut intuition. Even if I thought the investment might go up, I was still careful about not investing too much money. | 0.00 | 0.96 | 0.05 | 0.15 |
| 69 | Gen2Neg | Looked at the history of the returns on Asset B, and looked for patterns (i.e. periods that would rise or fall) that would help me make better decisions. | 0.00 | 0.76 | 0.25 | 0.80 |
| 70 | Gen2Neg | I think life for the most part is a dangerous game of chance. While it's not always dangerous to take some risks, better to be safe than sorry | 0.17 | 0.56 | 0.27 | 0.59 |
| 71 | Gen2Neg | I wrote down all the Asset B percentages and tried to look for patterns. For example, if I saw that I was getting high returns I knew there would only be a few more before it would go back into the negatives again. | 0.00 | 0.94 | 0.06 | 0.36 |
| 72 | Gen2Neg | Since I was told it was a large index fund, I figured that it would have a general upward trend, (unless I got stuck with the 1920s) so this lead me to invest more than I would normally be comfortable with into asset B | 0.04 | 0.84 | 0.12 | 0.59 |
| 73 | Gen2Neg | It was a matter of statistically evaluating returns and losses. If, after a period of high gains, followed by minimal gains, I knew that there was a higher likelihood of there being a crash, and vice versa. | 0.13 | 0.74 | 0.14 | -0.13 |
| 74 | Gen2Pos | When Asset B increase for 3-4 periods it will start decrease for 3 periods at least -25% then will goes up for 30% | 0.00 | 0.81 | 0.19 | 0.59 |
| 75 | Gen2Pos | Average rate helps me to get the allocation | 0.00 | 0.73 | 0.27 | 0.38 |
| 76 | Gen2Pos | I looked at 5 year patterns from previous returns and realized that Asset B's price stayed relatively constant for 3-4 years before it crashed at least 15%, so I made sure to keep most of my money in Asset A at all times while trying to safely win in the price boom periods. | 0.00 | 0.77 | 0.23 | 0.93 |
| 77 | Gen2Pos | Asset B's return would sink into the negative occasionally, but not too low. Also, the high returns were worth the risk as they were more common. | 0.16 | 0.65 | 0.20 | 0.08 |
| 78 | Gen2Pos | If I were to be given more information I may be able to better understand the future returns on Asset B. Since I know that Asset B is associated with large cap stocks since 1892 I know that the average return will be higher than 3%. It just depends on the time that you are selected in as to the average in the future. Past returns typically are not a great prediction of the future. | 0.04 | 0.86 | 0.10 | 0.56 |
| 79 | Gen2Pos | Because I had no historical data for the first several periods, I was simply making bets. After some periods, I began placing riskier bets after periods with negative returns and safer bets after periods with higher returns. | 0.19 | 0.75 | 0.06 | -0.67 |
| 80 | Gen2Pos | It was pretty up and down, and after it would get really high, it would typically drop | 0.10 | 0.74 | 0.16 | 0.27 |
| 81 | Gen2Pos | I thought that the stock had its ups and downs which was evident after I looked at the first few years. I then simply went more aggressive when it looked like Asset B was back to trending positively, and visa versa for when it had its negative trends I went less aggressive. | 0.11 | 0.75 | 0.14 | 0.36 |
| 82 | Gen2Pos | I formed my beliefs based on looking at the past returns for Asset B, and then going off of those numbers. | 0.00 | 0.89 | 0.11 | 0.36 |
| 83 | Gen2Pos | I form my beliefs based on the overall observation of assets B return--it starts to show a pattern after 4 periods, which makes it predictable. | 0.00 | 0.93 | 0.07 | 0.18 |
| 84 | Gen2Pos | I based my beliefs from a volatile perspective meaning there is a strong possibility that Asset B could crash or gain heavily. So I decided to never risk more than 35 percent of my money on Asset B at one time. | 0.05 | 0.68 | 0.26 | 0.87 |
| 85 | Gen2Pos | I'm aware of the stock market's historical performance and typically the market as a whole does fairly well. Since Asset B was based on some kind of index fund, I figured it would typically perform at least decently. Since I assumed it would pull positive most of the time, I just set a belief guess at 10% to encompass the positive values I assumed would be the most typical from 0-20%. | 0.00 | 0.82 | 0.18 | 0.93 |
| 86 | Gen2Pos | Over 15 to 20 years of asset return data I have convidence in the future of the us Stock market which is why by belief is optimistic | 0.00 | 0.84 | 0.16 | 0.59 |
| 87 | Gen2Pos | I based my decisions off of previous years. I also tried to look for patterns in the data to see if there was a way to guess fairly accurately what would happen, and towards the end I felt like that was working in my favor. | 0.00 | 0.89 | 0.11 | 0.64 |
| 88 | Gen2Pos | I mainly played it safe and kept to numbers ending in 5s and 10s. This allowed me to better track my own habits and compare it to how much money I made based on that. | 0.00 | 0.80 | 0.20 | 0.80 |
| 89 | Gen2Pos | I watched the previous trends and thought if it was going up, it would for a couple years, then it would usually max out and go down. There were some periods where it fluctuated heavily, which was unexpected | 0.00 | 1.00 | 0.00 | 0.00 |
| 90 | Gen2Pos | i just went around the previous return number, if it started to go down I invested less, if it went up I invested more etc. this often meant I missed out on the initial really high return rate but i also didnt lose too much too often. | 0.03 | 0.89 | 0.08 | 0.35 |
| 91 | Gen2Pos | It is very volatile | 0.00 | 1.00 | 0.00 | 0.00 |
| 92 | Gen2Pos | Based off of my results from the simulation | 0.00 | 1.00 | 0.00 | 0.00 |
| 93 | Gen2Pos | After a sudden down bit, it tended to go up fast at first then slowly rose to dip really fast again. | 0.00 | 1.00 | 0.00 | 0.00 |
| 94 | Gen2Pos | well a 3% return will keep up with inflation but will yield no dig returns to make money on them, how ever the are valuable in a ressetion. So asset B is the only real way to genarate returns. It is much risker, but more time than not it is positive. Time in the market will give the best results | 0.11 | 0.68 | 0.20 | 0.84 |
| 95 | Gen2Pos | Looking at the pattern of loses and winnings and making a guess | 0.15 | 0.63 | 0.22 | 0.30 |
| 96 | Gen2Pos | I took a similar approach to how I manage my Roth IRA. I was very risky with my portfolio and made it more conservative when I got closer to year 30. | 0.07 | 0.94 | 0.00 | -0.27 |
| 97 | Gen2Pos | Based on the fluctuation of the asset. | 0.00 | 0.71 | 0.29 | 0.36 |
| 98 | Gen2Pos | based on patterns but it was difficult because it kept shifting | 0.25 | 0.76 | 0.00 | -0.50 |
| 99 | Gen2Pos | It took me a couple of rounds to understand the trend of when it goes high it stays for about 2 turns and then it will go really low for a few turns | 0.07 | 0.93 | 0.00 | -0.34 |
| 100 | Gen2Pos | If I felt like I was on a "winning" streak (asset B % return greater than 0), I anticipated that I would soon receive a negative % return and would plan my next move accordingly. | 0.08 | 0.67 | 0.24 | 0.74 |
| 101 | Gen2Pos | I decided that although there would be some negatives there could possibly be more positives so I kept investing between 80-99 % because no matter what asset A was going to be 3% | 0.03 | 0.81 | 0.17 | 0.73 |
| 102 | Gen2Pos | I expected the returns to be within a standard deviation of the average return on Asset B. | 0.00 | 0.87 | 0.14 | 0.36 |
| 103 | Gen2Pos | it has big lows and highs so you need to keep your money longer so it will avarage on plus | 0.09 | 0.91 | 0.00 | -0.20 |
| 104 | Gen2Pos | Figuring out the actual percentage was trying to stay in the middle of the percentage that I predicted year before. Then doing high risk if the percentage was positive. | 0.06 | 0.83 | 0.11 | 0.36 |
| 105 | Gen2Pos | the returns variate but I think there is more chance of returns than risk | 0.15 | 0.68 | 0.17 | 0.07 |
| 106 | Gen2Pos | I formed my beliefs off previous returns and consistent return average from Asset A and Asset B. | 0.00 | 0.75 | 0.25 | 0.61 |
| 107 | Gen2Pos | I tried to look for a pattern if B went up multiple times a year I assumed a crash was coming. | 0.12 | 0.88 | 0.00 | -0.40 |
| 108 | Gen3NegPos | I based my beliefs on what Asset B was doing at the time after each belief question the average returns would differ for example there was one period where the average was around 15 percent with a lot of 20% gains and then after one of the questions the gains would drop to like 10 and start hitting negative returns more often. | 0.08 | 0.78 | 0.14 | 0.46 |
| 109 | Gen3NegPos | Every few years it would jump considerably and then descend just as quickly. | 0.00 | 1.00 | 0.00 | 0.00 |
| 110 | Gen3NegPos | based on how i could get the most money | 0.00 | 1.00 | 0.00 | 0.00 |
| 111 | Gen3NegPos | When putting returns on asset b, make sure to count in the negative from the previous return. | 0.16 | 0.62 | 0.21 | 0.03 |
| 112 | Gen3NegPos | Most of the time, the return rate was close to the last unless if dropped. I tried to keep track of whether I thought it was going to drop or stay steady up or down and made my assumptions based on that. | 0.05 | 0.95 | 0.00 | -0.27 |
| 113 | Gen3NegPos | Asset B would hardly go negative, sure it did but there were way more positives meaning profit. | 0.08 | 0.45 | 0.47 | 0.88 |
| 114 | Gen3NegPos | I observed how the average returns were very good and would most likely continue into the future | 0.00 | 0.83 | 0.17 | 0.49 |
| 115 | Gen3NegPos | I have no clue where this could go. The percentages changed so drastically you could never truly see where it was headed. Now, that's not to say you shouldn't invest in Asset B. Even if you lose a little bit in the bad years, the good years made up for it. | 0.21 | 0.74 | 0.05 | -0.84 |
| 116 | Gen3NegPos | As I went along I noticed that asset B most of the time will give me a great return so investing more leads to a greater return | 0.00 | 0.73 | 0.28 | 0.84 |
| 117 | Gen3NegPos | The stock market averages 18% so you might as well put all your money in it and let it average out at the end! | 0.00 | 0.91 | 0.09 | 0.34 |
| 118 | Gen3NegPos | It seemed like it was going to keep going up then there was a huge drop | 0.11 | 0.65 | 0.24 | 0.40 |
| 119 | Gen3NegPos | Asset B in the long run will go up. | 0.00 | 0.76 | 0.24 | 0.36 |
| 120 | Gen3NegPos | The return dramatically shifted within a large range of investments, and it got a little more manageable within a small range of investments. | 0.00 | 1.00 | 0.00 | 0.00 |
| 121 | Gen3NegPos | I watched how they kept moving and did similar predictions to the previous results | 0.00 | 1.00 | 0.00 | 0.00 |
| 122 | Gen3NegPos | I predicted that if Asset B were to exponentially increase it will soon level and then crash | 0.13 | 0.65 | 0.22 | 0.27 |
| 123 | Gen3NegPos | I tried to look for any kind of trends and kept track of how much the the current returns differed from the last period | 0.00 | 1.00 | 0.00 | 0.00 |
| 124 | Gen3NegPos | Asset B was too unpredictable. I never invested more than 30% of my money on asset B. If I had used my real money, I would not have invested in Asset B at all. | 0.00 | 0.81 | 0.20 | 0.76 |
| 125 | Gen3NegPos | I just would look at how it was growing and then decided an average on what i thought it would be around and then if i intuitively felt that it was going to be on a specific number based on past experience then i would put that actual number in as a guess. I started to realize that it was becoming very dangerous which then i would slowly guess the actual and put less allocation into it. | 0.04 | 0.91 | 0.05 | -0.27 |
| 126 | Gen3NegPos | I made my judgement based on the results of my decisions in the previous rounds of the exercise. | 0.00 | 1.00 | 0.00 | 0.00 |
| 127 | Gen3NegPos | I kept track of what asset B was at percentage wise and it was never consistant | 0.00 | 0.71 | 0.29 | 0.68 |
| 128 | Gen3NegPos | As I continued to make more money and based on previous years results, make small adjustments to keep a consistent increase and very little decrease. | 0.00 | 0.91 | 0.09 | 0.32 |
| 129 | Gen3NegPos | I tried to figure out a pattern but then I realized that there was no pattern. So then what I did was think to myself that not all good times last forever and not all bad times last forever. once I had a few good in a row, I would bring the percentage down and when there was 2 bad ones I would bring the percentages down. If I remember correctly I never, or almost never predicted to have a percentage under 0. | 0.11 | 0.81 | 0.08 | -0.47 |
| 130 | Gen3NegPos | I believe the future will match the returns I had in this game. There were good years and bad years. I didn't want the huge losses later in the game so I started moving some assets into safer options while still keeping assets in option be incase I could make more money | 0.15 | 0.70 | 0.15 | -0.07 |
| 131 | Gen3NegPos | There were more times where it went up a lot than down a lot | 0.00 | 1.00 | 0.00 | 0.00 |
| 132 | Gen3NegPos | I tried to figure out whether I had experienced the lowest returns to decide whether to keep investing larger amounts or not. | 0.11 | 0.89 | 0.00 | -0.38 |
| 133 | Gen3NegPos | Every decision I made was fully randomized. | 0.00 | 1.00 | 0.00 | 0.00 |
| 134 | Gen3NegPos | By looking at the data in the statement and trying to make educated guesses with all of the data | 0.00 | 1.00 | 0.00 | 0.00 |
| 135 | Gen3NegPos | I used past data | 0.00 | 1.00 | 0.00 | 0.00 |
| 136 | Gen3NegPos | I tried to follow rough trends and saw that after great returns or losses the market would often flip in a way and I tried t base my allocations off of this. | 0.07 | 0.82 | 0.11 | 0.34 |
| 137 | Gen3NegPos | asset B was entirely random there was no way to make an educated guess on it i did innitally think that this asset would be less volitale than it was but if you guessed the number 12 you were generally within +-10 of the actual value for the inital part of the experiment | 0.03 | 0.83 | 0.14 | 0.68 |
| 138 | Gen3NegPos | I believed that there was a trend that Asset b would be successful for two periods, followed by a dip. One of the prior comments stating there was a three year period of success made me feel that I should invest more after two periods of great success. | 0.00 | 0.71 | 0.29 | 0.96 |
| 139 | Gen3NegPos | I would have money in asset A and rarely put over 50% into Asset B but when I did, I made or lost a lot of money. | 0.10 | 0.79 | 0.12 | -0.12 |
| 140 | Gen3NegPos | I estimated my returns based on the pattern of returns for Asset B. | 0.00 | 0.83 | 0.17 | 0.36 |
| 141 | Gen3NegPos | I watched out for the decrease of asset B and how it effect asset a, based on probability | 0.00 | 0.76 | 0.24 | 0.61 |
| 142 | Gen3PosNeg | I tried to invest slow and steady. Sometimes I would make a big bet with a 10/90 distribution, but more often, I strayed towards the reliable 3% with 70% of my portfolio, and played the gambling game with the smaller 30% | 0.00 | 0.93 | 0.07 | 0.48 |
| 143 | Gen3PosNeg | I counted how many positive returns I got and weighed my odds against the negative, but for the future, I would say that the returns on asset B follow an upward trend. | 0.06 | 0.79 | 0.15 | 0.49 |
| 144 | Gen3PosNeg | I expected that it would never be negative twice and mainly just guessed upon that | 0.00 | 0.82 | 0.18 | 0.46 |
| 145 | Gen3PosNeg | I tried t guestimate where the percentage curve was gong to go and went with an average between the previous year and my guess-timation | 0.00 | 1.00 | 0.00 | 0.00 |
| 146 | Gen3PosNeg | I choose to stay safe and not rely on it. Putting only 30% or lower into it. | 0.11 | 0.75 | 0.14 | 0.18 |
| 147 | Gen3PosNeg | I know the average for SPY over the years so I took that into account. | 0.00 | 1.00 | 0.00 | 0.00 |
| 148 | Gen3PosNeg | Asset A doesn't give us a high enough return for anything substantial. Asset B is the only way you can get wealthy | 0.00 | 0.66 | 0.34 | 0.81 |
| 149 | Gen3PosNeg | I tried to see whether there was a pattern in my estimate based on the previous years | 0.00 | 1.00 | 0.00 | 0.00 |
| 150 | Gen3PosNeg | I looked for patterns and played it extra careful if I saw a big chance of negative returns, though I played it careful mostly the whole game. | 0.11 | 0.61 | 0.29 | 0.51 |
| 151 | Gen3PosNeg | I played it safe I did not want to put myself in a situation where I lost a lot of money | 0.14 | 0.66 | 0.21 | 0.42 |
| 152 | Gen3PosNeg | Asset B much more frequently had greater returns than Asset A, so I based my beliefs on the historical returns throughout the procedure | 0.00 | 0.72 | 0.28 | 0.78 |
| 153 | Gen3PosNeg | I treated it as a gamble. never put more than i was willing to lose | 0.16 | 0.84 | 0.00 | -0.40 |
| 154 | Gen3PosNeg | i base on return percentage in the last year and look some year before to check how circle work. | 0.00 | 1.00 | 0.00 | 0.00 |
| 155 | Gen3PosNeg | I tried to go off previous years. Even if I thought it would be successful I tried not to invest too much in case it went down. | 0.00 | 0.87 | 0.13 | 0.59 |
| 156 | Gen3PosNeg | I started with a base set of numbers and consistently added data then made conclusions off of this. I took risks when I knew I could and adjusted my numbers to the results. | 0.06 | 0.94 | 0.00 | -0.27 |
| 157 | Gen3PosNeg | They are good | 0.00 | 0.41 | 0.59 | 0.44 |
| 158 | Gen3PosNeg | When the return is significantly higher than average I become more cautious of a drop in returns. | 0.20 | 0.80 | 0.00 | -0.42 |
| 159 | Gen3PosNeg | I based mostly around the same range in which the returns were from the previous year. I would always estimate lower than the past return or high if it was in the negative. | 0.16 | 0.84 | 0.00 | -0.71 |
| 160 | Gen3PosNeg | Noticed that while it did have some large corrections it never really stayed in the red for all that long. | 0.00 | 1.00 | 0.00 | 0.00 |
| 161 | Gen3PosNeg | I tried to invest quite a bit in Asset B but the returns did not reward as much as I anticipated. | 0.16 | 0.77 | 0.07 | -0.50 |
| 162 | Gen3PosNeg | I really don't have any beliefs on how the future of asset B looks. The only conclusion I came to is that it can be volatile and relatively predictable. Each period brought more and more sporadic jumps, so I guess that would be its future? | 0.00 | 0.95 | 0.05 | 0.36 |
| 163 | Gen3PosNeg | always have some money in a, but b is worth investing in | 0.00 | 0.82 | 0.18 | 0.33 |
| 164 | Gen3PosNeg | After a couple periods of negative values I realized that there is going to be times when returns will not be a lot. | 0.14 | 0.77 | 0.10 | -0.25 |
| 165 | Gen3PosNeg | The trend was difficult to follow; down 5%, then 30%, then up 50%. Asset B is all over the place. Very volatile. | 0.10 | 0.80 | 0.10 | 0.00 |
| 166 | Gen3PosNeg | Previous year's return | 0.00 | 1.00 | 0.00 | 0.00 |
| 167 | Gen3PosNeg | The stock market seemed very volatile with a bearish trend in the middle. | 0.00 | 1.00 | 0.00 | 0.00 |
| 168 | Gen3PosNeg | I recognize that though there is great risk of losing it all in a crash, there is also great potential reward that is impossible by sticking to the risk free rate. | 0.20 | 0.48 | 0.32 | 0.83 |
| 169 | Gen3PosNeg | There would be some years where I would make a great amount of money back on Asset B, up to 30% back, but the next round the return would be -41%. It is very unpredictable and needs to be thought out carefully. | 0.00 | 0.87 | 0.13 | 0.62 |
| 170 | Gen3PosNeg | I just took into account the average we were told and went up a little because I was sure that the returns were greater with some outlying big losses bringing the average down. | 0.07 | 0.80 | 0.13 | 0.27 |
| 171 | Gen3PosNeg | They were extreme returns of either very low or high | 0.21 | 0.79 | 0.00 | -0.34 |
| 172 | Gen3PosNeg | For the majority of the years, the return of Asset B was positive. Therefore, I could see this trend continuing. | 0.00 | 0.75 | 0.25 | 0.73 |
| 173 | Gen3PosNeg | I just assumed I had 100 years of investing and looked at the data supplied on averages, and trusted the data so if this were prospective I would've assumed that everything would have fallen within the bounds of past performance, even though past performance doesn't indicate future results, history tends to repeat itself so trust the process. | 0.04 | 0.85 | 0.12 | 0.70 |
| 174 | Gen3PosNeg | I used past data along with the fact that it must converge to an average. It is a very volatile stock it seems, but its deviations are relatively equal | 0.00 | 1.00 | 0.00 | 0.00 |
| 175 | Gen3PosNeg | I would follow the trends in percentages of the previous year and i would base my next move either the same amount as the previous years return or i would go a little lower. | 0.06 | 0.95 | 0.00 | -0.23 |
| 176 | Gen3PosNeg | Consistent 20% returns will outweigh the losses from -40% dives in the market. | 0.18 | 0.82 | 0.00 | -0.40 |
| 177 | Gen3PosNeg | Kind of did a sort of card counting. I knew the average return was said to be 5% so when there was a few big losses in a row, I bet here and there. | 0.08 | 0.92 | 0.00 | -0.40 |
| 178 | Gen3PosNeg | I went based off the previous year's % of returns and evaluated from there. Although this simulation was run with a RNG, I believed that I saw some sort of fluctuating pattern that could help me identify what the future returns would be for the most part. | 0.00 | 0.95 | 0.06 | 0.40 |
| 179 | Gen3PosNeg | I tried to look for any patterns but every time I thought there was one, it changed. | 0.00 | 1.00 | 0.00 | 0.00 |
| 180 |  | when the assets decrease, they often continue to. in a steady slow down of decreases, you can expect a soon increase, and vice versa. sometimes there are sudden drops or hikes, however small calculated investments keep your negatives at a low loss and still get a fraction of the benefits if gone well. | 0.07 | 0.78 | 0.15 | 0.51 |
| 181 | Gen3PosPos | In general, I felt it was constantly around 15% from the average growth I was seeing. I wasn't thinking too much about growth or decline. | 0.00 | 0.82 | 0.18 | 0.64 |
| 182 | Gen3PosPos | I kept a tally of return rates and calculated an ongoing average. | 0.00 | 1.00 | 0.00 | 0.00 |
| 183 | Gen3PosPos | When i had a series of low percentages, I knew historically that not many years on indexes were low, therefore, once some low returns had passed I was more willing to allocate more to asset B. | 0.15 | 0.78 | 0.07 | -0.37 |
| 184 | Gen3PosPos | putting your money in the market and not guessing year to year is the best way to garuntee a positive return. | 0.00 | 0.71 | 0.29 | 0.83 |
| 185 | Gen3PosPos | The rate of return is so fluctuating if you play your cards right you will make a lot of money. Worth the risk. | 0.08 | 0.76 | 0.16 | 0.30 |
| 186 | Gen3PosPos | I'll take the risk on asset B, because it only decreased 9 times in total | 0.12 | 0.74 | 0.14 | 0.10 |
| 187 | Gen3PosPos | Asset B is high risk which means high return but it can be unpredictable | 0.10 | 0.78 | 0.11 | 0.05 |
| 188 | Gen3PosPos | I formed my beliefs regarding the future returns on Asset B because as I started the simulation the odds of you losing money on Asset B in the beginning were very low compared to the end of the simulation. | 0.11 | 0.78 | 0.11 | 0.00 |
| 189 | Gen3PosPos | I estimated the future returns by reviewing the current returns and guessing. Also, I know that if there is a couple bad years then that means it will usually start going back up after a couple of down years. | 0.08 | 0.92 | 0.00 | -0.54 |
| 190 | Gen3PosPos | i started off investing s lot with high returns because i believed it would get you to trust it then it crash | 0.10 | 0.77 | 0.13 | 0.15 |
| 191 | Gen3PosPos | I recognized 20-30% was the most common percentages and stuck in that range even through dips | 0.12 | 0.88 | 0.00 | -0.25 |
| 192 | Gen3PosPos | Going off the past and averages given. | 0.00 | 1.00 | 0.00 | 0.00 |
| 193 | Gen3PosPos | I know that markets typically increase year over year so I continually invested a majority of my money in Asset B. | 0.00 | 0.80 | 0.20 | 0.59 |
| 194 | Gen3PosPos | Though i lost a good amount of money during a couple of the periods, because I had been so successful early on, it allowed me to quickly recover my loss and not "feel it." | 0.11 | 0.71 | 0.18 | 0.63 |
| 195 | Gen3PosPos | Asset B would maintain positive returns for a few rounds. The longer these rounds of positive returns, the higher a chance of a fall, say around 3 confident turns. The falls were unpredictable and I didn't try to predict them. Instead, I focused on predicting the positive returns, usually around the 20% mark, with a lot of room for deviation. I estimated on the low side because if it exceeds my prediction, I still win in payout. | 0.02 | 0.72 | 0.26 | 0.97 |
| 196 | Gen3PosPos | There were hardly any negative results; a couple just below breaking even and a couple big negative years. For the most part I thought the gains from Asset B were usually greater than the 3% ROI from Asset A so I consistently put more than 50% of my portfolio into asset B | 0.11 | 0.70 | 0.19 | 0.51 |
| 197 | Gen3PosPos | It is a risky investment. one should only invest but a little percentage of their money in this asset | 0.06 | 0.77 | 0.17 | 0.53 |
| 198 | Gen3PosPos | I've personally experienced investing in the market before, I also just used the statistics given to us in the pamphlet | 0.00 | 1.00 | 0.00 | 0.00 |
| 199 | Gen3PosPos | it just how i was raised with my family | 0.00 | 1.00 | 0.00 | 0.00 |
| 200 | Gen3PosPos | The market was strong through the first two periods. I should have rode the upward trend. | 0.00 | 0.82 | 0.18 | 0.51 |
| 201 | Gen3PosPos | there were few times it went down and even when you lost money it always came back in the end | 0.11 | 0.89 | 0.00 | -0.32 |
| 202 | Gen3PosPos | My beliefs were based on the average of asset B | 0.00 | 0.78 | 0.22 | 0.36 |
| 203 | Gen3PosPos | US Stocks have historically gone up, so I allocated lots to B because S&P yields 8% annualized over last 50ish years. | 0.00 | 1.00 | 0.00 | 0.00 |
| 204 | Gen3PosPos | at first I wasn't sure what they were talking about but the more I did the experiment the more I understood that asset B made money but became risky at the end | 0.10 | 0.81 | 0.09 | 0.15 |
| 205 | Gen3PosPos | I was a little bit confused at first and played it safe to begin but slowly got the hang of it. I did not go very high. | 0.05 | 0.82 | 0.13 | 0.28 |
| 206 | Gen3PosPos | i saw it was mostly positive along with what info we were told so i went all in on it for the most part and earned an extra 10 dollars | 0.00 | 0.89 | 0.11 | 0.56 |
| 207 | Gen3PosPos | Well taking in with what the others said about how good it is I thought what could go wrong and for the most part it worked, I only really got bit a few times. | 0.08 | 0.79 | 0.13 | 0.23 |
| 208 | Gen3PosPos | Asset B is somewhat similar to the S&P 500, granted the returns are very high sometimes but generally there is a positive average return, because of this, I went with a high allocation of investments to B. I believe over the long run it would always go up. | 0.00 | 0.85 | 0.15 | 0.80 |
| 209 | Gen3PosPos | I figured that if it was way up it had top come down and vis versa. Therefore I always kept my amounts roughly around the -9 to 19 range. | 0.00 | 0.94 | 0.06 | 0.20 |
| 210 | Gen3NegNeg | Asset B is risky investment but it has potential too. | 0.13 | 0.72 | 0.16 | 0.09 |
| 211 | Gen3NegNeg | Looking at the numbers, I knew investing a bit more into B was going to go in my favor, but not too much where I had nothing for A | 0.00 | 0.94 | 0.06 | 0.21 |
| 212 | Gen3NegNeg | I believe Asset B is unpredictable and must be approached with caution. | 0.00 | 0.82 | 0.19 | 0.36 |
| 213 | Gen3NegNeg | The instructions said that the average over all time for asset B was 5%, which was greater than asset A's 3%, and while the returns on asset A varied radically, I knew that over time it would yield a return greater than asset B. | 0.00 | 0.72 | 0.28 | 0.92 |
| 214 | Gen3NegNeg | Every time when the market plans to crush, the government or big money will help to prevent happen. The capital is greedy but also is learning to avoid big economy crisis. Capital is serving for the politics and is the game of big financial groups. | 0.21 | 0.73 | 0.05 | -0.86 |
| 215 | Gen3NegNeg | Based on the risk factor from experience and historical data | 0.19 | 0.81 | 0.00 | -0.27 |
| 216 | Gen3NegNeg | the return of the last year went up, I will increase my belief on the Asset B. | 0.00 | 0.76 | 0.24 | 0.59 |
| 217 | Gen3NegNeg | I found the returns to be somewhat arbitrary and unpredictable. This was based on the fact that some years, there was a large return, whereas in others, there was a large loss, and no apparent rhyme or reason. | 0.11 | 0.89 | 0.00 | -0.54 |
| 218 | Gen3NegNeg | I kept seeing the patterns in the numbers. How It would have negative returns every couple years or so. I would be careful after a super high reward of over 100,000 and would not make the same calculation the year after that as it would end up being negative or a lower number that would cause me to lose money. I just tried to see if there was a pattern on every couple years having crashes and then following that it would go back up and then the numbers would go down again. | 0.11 | 0.79 | 0.10 | -0.42 |
| 219 | Gen3NegNeg | Some times the returns were high yet there were also some negative returns to watch out for | 0.19 | 0.81 | 0.00 | -0.57 |
| 220 | Gen3NegNeg | Most of my beliefs were based on the statistics box given to us in the instructions. I also thought about periods in history with major market crashes. | 0.00 | 1.00 | 0.00 | 0.00 |
| 221 | Gen3NegNeg | I made my estimates within a close range not too far from the previous year | 0.00 | 1.00 | 0.00 | 0.00 |
| 222 | Gen3NegNeg | In order to make big investments, you got to take big risks, and thats what I believed in Asset B | 0.09 | 0.80 | 0.11 | 0.10 |
| 223 | Gen3NegNeg | Never knowing what the returns might be like is why I decided to play it safe. There were times where if I invested it all I would have made a lot, but the risk did not outweigh the benefit. Risking it all would have put me in a far worse position. | 0.21 | 0.71 | 0.09 | -0.86 |
| 224 | Gen3NegNeg | I tried to look at the patterns based on the data I had towards the end. In the beginning I tried to base my information off of the previous average and standard deviation that we were given. I learned that it was better to not rely on Asset B to follow the trends in a consistent way. | 0.04 | 0.92 | 0.05 | 0.20 |
| 225 | Gen3NegNeg | I think it is too risky to put most of your money on asset B | 0.10 | 0.75 | 0.15 | 0.18 |
| 226 | Gen3NegNeg | Asset B has its times of high returns and you never know when it will fall. only invest what you are willing to loose. | 0.09 | 0.82 | 0.09 | 0.05 |
| 227 | Gen3NegNeg | There were only a few instances where the percent return on Asset B were very low. In most cases the percent return was above 10% so I felt that it was usually safe to put money in Asset B. | 0.05 | 0.77 | 0.17 | 0.67 |
| 228 | Gen3NegNeg | It's clear to see that Asset B is more volatile in the experiment, and as such I didn't put a lot into it. | 0.00 | 0.81 | 0.20 | 0.62 |
| 229 | Gen3NegNeg | In the beginning it was trail and error but after some time I noticed when the percentages were high (30%) I could expect a drop. Towards the end I noticed the stock became more volatile which made me invest less and expect random numbers. | 0.10 | 0.90 | 0.00 | -0.54 |
| 230 | Gen3NegNeg | I try to invest when it crashed and put money into Asset A when it got too high for comfort (Around 24%) | 0.00 | 0.80 | 0.20 | 0.61 |
| 231 | Gen3NegNeg | The returns are always very unexpected | 0.00 | 1.00 | 0.00 | 0.00 |
| 232 | Gen3NegNeg | I kind of went with consistence. If B kept being higher that 3% then I would put more in B. I only put some in A in case B dropped a lot. | 0.00 | 1.00 | 0.00 | 0.00 |
| 233 | Gen3NegNeg | After losing lots of money | 0.39 | 0.61 | 0.00 | -0.38 |
| 234 | Gen3NegNeg | It seemed risky, so I never invested more than 50% in it. It would give me a big return but as soon as I increased my investment, it would drop. | 0.12 | 0.80 | 0.08 | -0.10 |
| 235 | Gen3NegNeg | Asset B is always valuable even if you lose in one year. If you invest 25% even if you have a negative return you still make some gains from A and therefore there isnt much risk in allocating to B | 0.13 | 0.68 | 0.20 | 0.34 |
| 236 | Gen3NegNeg | i was trying to be consistent with my numbers | 0.00 | 1.00 | 0.00 | 0.00 |
| 237 | Gen3NegNeg | I based my strategy off of the advice prior people gave. All the subjects on the paper said Asset B was risky and you could loose a lot of money on it. I would rather see a consistent flow of income (Asset A) rather than risking my savings on Asset B. With compounding interest my account was starting to go up, that is why I never invested into Asset B. | 0.11 | 0.77 | 0.13 | 0.46 |
| 238 | Gen3NegNeg | I would give a general estimation on trend of Asset B before I put in my number. And even then I would aim to pick a more general number such as 10 because I could get points if it went up to 20 or down to 0 where there was a higher chance of the returns being in those brackets. | 0.00 | 0.88 | 0.12 | 0.66 |
| 239 | Gen3NegNeg | Asset B averaged around 10%, but went all the way down to -30% at one point. So, I was cautious when choosing to put my money in there. I stayed below 50 for almost every decision. | 0.04 | 0.91 | 0.05 | 0.04 |
| 240 | Gen3NegNeg | i watched the increasing rate and decreasing rate as inflation would rapidly change due to asset B having a high value | 0.00 | 0.80 | 0.21 | 0.60 |
| 241 | Gen3NegNeg | I formed my beliefs by investing only small amounts of money. It gained me a good amount and when a giant dip came I did not lose all of my money. | 0.00 | 0.78 | 0.22 | 0.78 |
| 242 | Gen3NegNeg | Asset B had volatile returns. Some periods would be tremendous and when you started to believe in the return, it would tank. | 0.00 | 0.89 | 0.11 | 0.36 |
| 243 | Gen3NegNeg | There's a love/hate relationship with Asset B. Sometimes it's good, but most of the time I stopped investing so much into it. | 0.09 | 0.76 | 0.15 | 0.09 |
| 244 | Gen3NegNeg | Based on the actual return, I kept about 10 within that | 0.00 | 1.00 | 0.00 | 0.00 |
| 245 | Gen3NegNeg | I completely guessed | 0.00 | 1.00 | 0.00 | 0.00 |
| 246 | Gen3NegNeg | My beliefs were that every so often I could risk some money on Asset B if I knew there would be enough at the end. For myself I did no more than 40 due to wanting to keep my investment growing in Asset A. | 0.09 | 0.78 | 0.13 | 0.34 |
| 247 | Gen3NegNeg | Although it is very spiritic, asset B seems to remain positive more often than negative. However, it still has sudden unexpected crashes and is an unwise place to allocate most resources. | 0.10 | 0.73 | 0.17 | 0.34 |
| 248 | Gen3NegNeg | Starting the experiment I wasn't sure. I had very high feelings towards asset B before the 2nd survey question, it seemed as if every time asset B was returning a positive outcome, after that 2nd time however I got hit with a -41% and that drastically shifted my opinion more against the asset. | 0.03 | 0.78 | 0.19 | 0.86 |

**Appendix VI: Regression Results Experiment 2**

We start by considering the effects on stock allocations of participants in the Negative/Negative advice treatment compared to Positive/Positive advice treatment. The effects are summarized in Table A6.1.

As seen in Table A6.1, subjects who received negative advice from both prior generations held, on average, between 17 and 23 percentage points less in stocks than subjects in Generation 3 who received positive from both prior generations, after controlling for wealth effects, age, sex, education and investment experience in all regression specifications, bar for the last one. In the last column, FRM, the fractional response model shows that subjects who received negative advice from both generations reduced their share of stocks by 44% of their entire portfolio, relative to subjects who received positive advice twice, a massive difference. More experience in investing was associated with more stock holdings in all regression specifications. Sex was generally statistically insignificant (except in Prais-Winsten and FRM regressions) but when it was significant, males held more stocks than females, a result that is in line with a large literature on sex and risk aversion within the financial domain. Education was generally statistically insignificant because the differences in education levels were quite small, as the overwhelming majority of the sample were college students. But it is worth noting that in two econometric specifications subjects in Generation 3 with more education held less stocks (in the Prais-Winsten and FRM regressions). Finally, it is also worth noticing that wealth

**Table A6.1 – Regression Analyses of Portfolio Allocations for Experiment 2**

Dependent Variable is Stocks

|  | (1) | (2) | (3) | (4) | (5) | (6) |
| --- | --- | --- | --- | --- | --- | --- |
| VARIABLES | OLS | Tobit | Auto | RE | MLM | FRM |
| Gen 3 Negative-Negative | -16.61*** | -17.79*** | -16.61*** | -21.56*** | -22.45*** | -0.444*** |
|  | (4.786) | (5.388) | (1.601) | (5.864) | (5.944) | (0.0367) |
| Gen 3 Positive-Negative | -7.085 | -7.610 | -7.085*** | -9.689 | -10.04* | -0.190*** |
|  | (4.624) | (5.334) | (1.299) | (5.899) | (5.897) | (0.0367) |
| Gen 3 Negative-Positive | -7.692* | -9.269* | -7.692*** | -10.29* | -10.77* | -0.208*** |
|  | (4.581) | (5.118) | (1.151) | (5.867) | (6.021) | (0.0368) |
|  |  |  |  |  |  |  |
| Beginning Account Balance (Lag 1) | 6.49e-05*** | 7.47e-05*** | 6.49e-05*** | 4.31e-06 | -7.23e-06*** | 1.78e-06*** |
|  | (6.79e-06) | (8.65e-06) | (1.64e-05) | (4.22e-06) | (2.10e-06) | (7.81e-08) |
| Age | 0.386 | 0.360 | 0.386*** | 0.459 | 0.474 | 0.00995*** |
|  | (0.416) | (0.403) | (0.0475) | (0.520) | (0.419) | (0.00233) |
| Sex | 5.402 | 5.450 | 5.402*** | 6.864 | 7.871* | 0.142*** |
|  | (3.629) | (3.833) | (0.685) | (4.522) | (4.257) | (0.0241) |
| Education | -2.939 | -3.063 | -2.939*** | -3.380 | -3.596* | -0.0773*** |
|  | (1.844) | (2.011) | (0.239) | (2.328) | (2.163) | (0.0126) |
| Experience | 3.285 | 3.703 | 3.285*** | 4.325* | 4.498* | 0.0878*** |
|  | (2.066) | (2.323) | (0.412) | (2.627) | (2.521) | (0.0146) |
|  |  |  |  |  |  |  |
| Constant | 25.79*** | 23.94** | 25.79*** | 42.86*** | 46.08*** | -0.646*** |
|  | (9.166) | (9.917) | (5.365) | (11.42) | (11.13) | (0.0656) |
|  |  |  |  |  |  |  |
| Observations | 4,118 | 4,118 | 4,118 | 4,118 | 4,118 | 4,118 |
| R-squared | 0.228 |  | 0.228 |  |  |  |
| Number of groups |  |  |  |  | 142 |  |
| Number of subjects |  |  | 142 | 142 |  |  |

Robust standard errors in parentheses (std. errors clustered by subject except for (5), which is clustered by condition)

*** p<0.01, ** p<0.05, * p<0.1

**Table A6.2 – Regression Analyses of Risk Aversion for Experiment 2**

Dependent Variable is Risk-Aversion

|  | (1) | (2) | (3) | (4) | (5) |
| --- | --- | --- | --- | --- | --- |
| VARIABLES | OLS | Tobit | Auto | RE | MLM |
| Gen 3 Negative-Negative | 0.417** | 0.417** | 0.417*** | 0.415** | 0.418** |
|  | (0.180) | (0.180) | (0.0289) | (0.179) | (0.172) |
| Gen 3 Positive-Negative | 0.578*** | 0.578*** | 0.578*** | 0.577*** | 0.578*** |
|  | (0.177) | (0.177) | (0.0252) | (0.177) | (0.174) |
| Gen 3 Negative-Positive | 0.403** | 0.403** | 0.403*** | 0.402** | 0.403** |
|  | (0.187) | (0.187) | (0.0211) | (0.187) | (0.178) |
| Beginning Account Balance (Lag 1) | -6.99e-07*** | -6.99e-07*** | -6.99e-07*** | -7.20e-07*** | -6.93e-07*** |
|  | (2.53e-07) | (2.53e-07) | (1.84e-07) | (2.24e-07) | (7.70e-08) |
| Age | 0.0206** | 0.0206** | 0.0206*** | 0.0206** | 0.0205* |
|  | (0.00948) | (0.00947) | (0.00122) | (0.00948) | (0.0122) |
| Sex | -0.407*** | -0.407*** | -0.407*** | -0.407*** | -0.407*** |
|  | (0.106) | (0.106) | (0.0238) | (0.106) | (0.123) |
| Education | 0.0672 | 0.0672 | 0.0672*** | 0.0670 | 0.0668 |
|  | (0.0596) | (0.0595) | (0.0134) | (0.0595) | (0.0630) |
| Experience | 0.0769 | 0.0769 | 0.0769*** | 0.0772 | 0.0776 |
|  | (0.0756) | (0.0755) | (0.0164) | (0.0757) | (0.0735) |
| Constant | 2.291*** | 2.291*** | 2.291*** | 2.297*** | 2.291*** |
|  | (0.314) | (0.313) | (0.0743) | (0.307) | (0.324) |
|  |  |  |  |  |  |
| Observations | 4,118 | 4,118 | 4,118 | 4,118 | 4,118 |
| R-squared | 0.126 |  | 0.126 |  |  |
| Number of groups |  |  |  |  | 142 |
| Number of subjects |  |  | 142 | 142 |  |

Robust standard errors in parentheses (std. errors clustered by subject except for (5), which is clustered by condition)

*** p<0.01, ** p<0.05, * p<0.1

effects were statistically associated with more stock holdings, but the effect was quantitatively too small to be economically meaningful.

Next, as in Experiment 1, we examined if the channel of transmission from advice to stock allocations went through beliefs and/or risk attitudes. Was the advice effect implemented by making subjects receiving reinforcing advice either more optimistic or more pessimistic? The brief answer is “NO”. When we run regressions with “belief” as the dependent variable, mirroring results presented in Table 6 for Experiment 1, results came up statistically insignificant in all regression specifications, except for the Prais-Winsten regression specification. Therefore, in this case, it does not seem that expectations about future stock market returns play a role in explaining subjects’ stock allocations. We make these results available upon request.

Figure 10 graphs the belief forecast held by participants in the Negative/Negative and Positive/Positive treatments. A few things are clarified by the information provided by Figure 10/Table A6.3. First, note that in years 1-3, the average beliefs reported by subjects in the Negative/Negative treatment are higher than the average beliefs of subjects in the Positive-Positive treatment (18%>16%). For whatever reason, subjects in the Negative-Negative condition started off the experiment with more optimistic beliefs than subjects in the Positive-Positive condition. But across the entire 30 periods of the investment task, the average beliefs of the two cohorts are not significantly different (Pos/Pos=15%, Neg/Neg=13%).

On the other hand, Table A6.3 shows that the reported risk aversion in both sub-cohorts is indeed different. Note how the reported risk aversion of subjects in the Negative-Negative condition is always above the value “3” in all subperiods whereas for subjects in the positive-positive condition the reported risk aversion is, for all sub-periods, below the value “2”. In fact, a t-statistic test of difference in means shows that the difference in reported risk aversion is in fact statistically significant. Risk aversion seems to act as a relevant transmission channel.

To confirm the role of risk aversion as a relevant transmission channel we present regressions in which risk aversion is the dependent variable run against the set of right-hand side controls used prior regressions. Table A6.2 summarizes those results.

***---Insert Table 10 about here---***

To avoid over-loading subjects with non-essential tasks to be performed every period, risk aversion was only reported by subjects every 10 periods. Therefore, in order to be able to run regressions with year-by-year variation a number of procedures were tried, none of them ideal, but all of them sensible. First, we assumed that the reported risk aversion stayed constant between periods when subjects self-reported their risk aversion. This is our first proxy variable for year-by-year risk aversion (ra1). Next, we tried linear interpolation to add more variation to the risk aversion proxy variable over the years (ra2). Next, we tried non-linear interpolation via polynomial algorithms (a quadratic polynomial was our ra3). Finally, we used the inverse of the portfolio standard deviation multiplied by the share of stocks held by each subject each period and multiplied the resulting expression by an index number that mapped the account balance of each subject each period one-for one, so as to reflect risk aversion increasing one-for-one with every extra dollar in the subjects’ account balance (this was our ra4). All the proxies are problematic in their own specific ways. All but ra1 created severe econometric problems with some of the estimation specifications and had to be dropped. We settled for ra1 as the best compromise even when in some cases it is clear it does not provide the best regression fit.

Results displayed in Table A6.2 show, the relevant dummy variable (Gen 3 Negative-Negative) is statistically significant at least at 5% in all specifications, after controlling for age, sex, education and financial experience. It is worth noting too that in the regression specification that controls for autocorrelation (Prais-Winsten), the significance level is 5%. Once we consider that risk aversion only had time variation every 10 years, this result is not bad and fully in line with the statistical result summarized in Table A6.3. Also worth noting is that males were significantly less risk-averse than females in Experiment 2. Also, at the 5% level of statistical significance, subjects who received mixed advice were less risk-averse than subjects who received positive advice twice in a row.

|  |  | Table A6.3, Experiment 2 | | |  |  |
| --- | --- | --- | --- | --- | --- | --- |
| Generation 3 Pos Pos | AVG | AVG | AVG | Median | Median | Median |
|  | Stocks | Belief | RA | Stocks | Belief | RA |
| YR1 | 56.97 | 0.15 | 2.97 | 60.00 | 0.09 | 3 |
| Yrs 1-3 | 59.48 | 0.16 | 2.97 | 65 | 0.15 | 3 |
| YRs 4-30 | 59.02 | 0.15 | 2.61 | 70 | 0.12 | 3 |
| YRs 10-30 | 59.59 | 0.15 | 2.51 | 70 | 0.11 | 2 |
| AVG Yrs 1-30 | 59.06 | 0.15 | 2.65 | 70.00 | 0.12 | 3 |
| Generation 3 Neg Neg | AVG | AVG | AVG | Median | Median | Median |
|  | Stocks | Belief | RA | Stocks | Belief | RA |
| YR1 | 34.20 | 0.13 | 3.41 | 60.00 | 0.09 | 3.00 |
| Yrs 1-3 | 35.30 | 0.18 | 3.41 | 30 | 0.15 | 3 |
| YRs 4-30 | 33.88 | 0.13 | 3.12 | 25 | 0.1 | 3 |
| YRs 10-30 | 33.67 | 0.12 | 3.03 | 25 | 0.1 | 3 |
| AVG Yrs 1-30 | 34.03 | 0.13 | 3.15 | 25.00 | 0.10 | 3.00 |
| Differences in means | Stocks | Belief | RA |  |  |  |
| Yrs 1-30 | -25.34 | -0.02 | 0.52 |  |  |  |
| StdError | 0.90 | 0.05 | 0.10 |  |  |  |
| t-stat of diff. | 27.92*** | 3.17*** | 5.28*** |  |  |  |
