# Supplementary material for: An experiment on the impacts of experiential investment advice
Source: PLoS One. 2026 Feb 20;21(2):e0343126. doi: 10.1371/journal.pone.0343126 (PMC12923020; doi:10.1371/journal.pone.0343126)
Supplement: Attachment — Submitted filename: Response_to_Reviewers_auresp_2.docx [file pone.0343126.s005.docx]

Review Comment: I am willing to accept the current version of the paper as you convincingly addressed the reviewers' and my concerns. However, please carefully recheck the paper (+ supplemental part) for spelling errors. For example, I picked up "S2 Supplemnetal...

- *Response: We thank the editor for the careful reading of our paper. We have rechecked and corrected the paper and supplements S1 and S2 for spelling errors and edits.*
